# Supplementary material for: Evaluation of an online suicide prevention program to improve suicide literacy and to reduce suicide stigma: A mixed methods study
Source: PLoS One. 2023 Apr 28;18(4):e0284944. doi: 10.1371/journal.pone.0284944 (PMC10146514; doi:10.1371/journal.pone.0284944)
Supplement: S1 Table — (PDF) [file pone.0284944.s001.pdf]

S1 Table. Content of the online program and variants

The developed online program contained eight chapters (see table S1a) including several short video sequences of eight persons sharing their lived experiences of suicide, including helpful experiences in dealing with own suicidality or the suicide of a close person. The focus of these video reports was to convey hope, to show how to talk about the topic of suicidality and suicide, and to point out support options in crises. The program also contained evidence-based fact sheets on suicidality, exercises based on cognitive-behavioral models, and worksheets. A help section was always visible during the program providing external professional support services via online links and telephone numbers of national and regional services, crisis lines and locations of emergency mental health services. The program development closely involved ten persons with lived experiences of suicide ("lived experience team").

Table S1a. *Content of the online suicide prevention program "8 lives – lived experience reports and facts on suicide" [German title "8 Leben – Erfahrungsberichte und Wissenswertes zum Thema Suizid"]*

**Chapter 1: Demographics ("*About me*")**
– Age, gender, level of education, size of residence, own experience with suicidality or suicide
**Chapter 2: Baseline assessment t_0 ("*My thoughts*")**
– Primary and secondary outcome assessment
**Chapter 3: Psychoeducation ("*My knowledge*")**
– Evidence-based health information: meaning of suicide and suicidality, possible causes of suicidality, warning signs, precipitating events, risk and protective factors; suicidality as a continuum
**Chapter 4: Experience reports on suicidality ("*My story*")**
– Video reports and text messages by persons with a lived experience of suicide
– Suicide taboo and suicide stigma; falsities concerning suicidality as opposed to reality
– Possibility to anonymously communicate own experiences with suicidality or suicide ("digital postcard")
**Chapter 5: Strategies I - Behavior, Mind, Body, Feelings ("*My coping*")**
– Introduction of the concept of a safety plan in case of suicidality
– Link between activity and well-being, creating a personal list of positive activities; cognitive restructuring technique; progressive muscle relaxation; psychoeducation about feelings [all optional]
**Chapter 6: Strategies II - Communication ("*Exchange*")**
– Strategies for communication with different groups of people (family, friends, physicians, psychotherapists)
– Reflection on the disclosure of suicidality or suicide
**Chapter 7: Personal goal setting ("*My goals*")**
– Personal goal setting according to "SMART" criteria regarding the strategies presented in Chapter 5 and 6
**Chapter 8: Post assessment t_1 ("*Feedback*")**
– Primary and secondary outcome assessment; feedback on the program (satisfaction, helpful elements)

Note. The content and development process of the online program are in detail described in: Dreier, M., Baumgardt, J., Bock, T., Härter, M., The 8 Lives Team, & Liebherz, S. (2021). Development of an online suicide prevention program involving people with lived experience: ideas and challenges. Research Involvement and Engagement, 7, 60 https://doi.org/10.1186/s40900-021-00307-9

Depending on the self-reported type of affectedness, participants were assigned to one of five program variants (see table S1b). The content (e.g., texts, videos of persons with a lived experience of suicide, work sheets) varied accordingly.

Table S1b. *Five variants of the online suicide prevention program 8 lives*

	Program variant	Kind of suicide experience
**Affected**	1	Participant has or had suicidal ideation.
	2	Participant made one or more suicide attempts.
**Affected as a close person**	3	Participant lost a close person by suicide.
	4	Participant is caring for a suicidal person close to him/her.
**Interested/other**	5	Participant has a general interest in the topic of suicidality, e.g., as a health care professional, or is otherwise affected by the topic.
