# Supplementary material for: Evaluation of an online suicide prevention program to improve suicide literacy and to reduce suicide stigma: A mixed methods study
Source: PLoS One. 2023 Apr 28;18(4):e0284944. doi: 10.1371/journal.pone.0284944 (PMC10146514; doi:10.1371/journal.pone.0284944)
Supplement: S2 Table — (PDF) [file pone.0284944.s002.pdf]

S2 Table. Web analytics

The actual cumulative time spent on the eight chapters of the online program, the number of visitors, and the average time spent on the website were recorded using the open-source web analytics tool Matomo (https://matomo.org/).

Using Matomo, the program's homepage (https://8leben.psychenet.de/) recorded 7,369 unique visits during the nine-month survey period. On average, persons stayed for 54 seconds on the homepage. Persons who logged in the program, i.e. study participants, showed a total processing time for eight chapters of 69.6 minutes on average (see table S2).

Table S2. *Cumulative time spent on the eight chapters and the number of visitors measured by the web analytics tool Matomo.*

Chapter	Average time spent in minutes	Number of visitors
**1**	2.2	701
**2**	5.4	714
**3**	16.2	787
**4**	17.0	557
**5**	11.8	404
**6**	10.2	297
**7**	2.4	286
**8**	4.5	284
**Total**	69.6	/

Note. The web analytics tool Matomo uses aggregated data so that non-completers are also considered in the average time spent in each chapter. Only participants who agreed to use the web analytics tool Matomo are included. As a registered participant it was possible to visit the chapters several times.
