# Supplementary material for: Evaluation of an online suicide prevention program to improve suicide literacy and to reduce suicide stigma: A mixed methods study
Source: PLoS One. 2023 Apr 28;18(4):e0284944. doi: 10.1371/journal.pone.0284944 (PMC10146514; doi:10.1371/journal.pone.0284944)
Supplement: S4 Table — (PDF) [file pone.0284944.s004.pdf]

S4 Table. Consolidated criteria for reporting qualitative studies (COREQ)

According to COREQ checklist (Tong et al., 2007), we described the items in the table below. If an item did not apply to our study, we indicate N/A. If we described the item in the manuscript, we entered the section.

Table S4. Consolidated criteria for reporting qualitative studies (COREQ): 32-item checklist developed by (Tong et al., 2007)

No	Item	Description or section in the manuscript
**Domain 1: Research team and reflexivity**		
Personal Characteristics		
1.	Interviewer/facilitator	MD [interviewer, evaluator]<br>JB [evaluator]<br>SL [supervisor, principal investigator]<br>NP [advisor]
2.	Credentials	MD: Dipl.Psych. corresponding to M.Sc. Psychology<br>JB: Social scientist, PhD<br>SL: Licensed clinical psychologist (CBT), PhD<br>NP: Psychologist, PhD
3.	Occupation	MD: Research assistant at Department of Medical Psychology, University Medical Center Hamburg-Eppendorf, Hamburg, Germany<br>JB: Research assistant at Department of Psychiatry and Psychotherapy, Center for Psychosocial Medicine, University Medical Center Hamburg-Eppendorf, Hamburg, Germany<br>SL: Head of research group at Department of Medical Psychology, University Medical Center Hamburg-Eppendorf, Hamburg, Germany<br>NP: Research assistant at Department of Primary Medical Care, University Medical Center Hamburg-Eppendorf, Hamburg, Germany
4.	Gender	Female
5.	Experience and training	*What experience or training did the researcher have?*<br>MD (interviewer and evaluator): Psychologist and research assistant. Research assistant in one qualitative interview study before this study; she participated in a qualitative method seminar at university medical center. MD is in training as a licensed specialist in psychological psychotherapy. To increase intersubjective reproducibility and comprehensibility, MD presented and discussed the qualitative research twice with an interdisciplinary group of researchers in a qualitative methods seminar led by NP.

No	Item	Description or section in the manuscript
		JB (second evaluator) is a social scientist, has a PhD, and has been researching various topics in social psychiatric care research for several years. She brings personal experience to the "8 Lives" project, having lost her father by suicide at the age of 21.<br><br>SL (supervisor, principal investigator): Licensed specialist in psychological psychotherapy (CBT).<br><br>NP (advisor): Supervisor of a qualitative method seminar at University Medical Center Hamburg-Eppendorf, post-doc psychologist, experienced qualitative researcher (interviews, focus-groups, qualitative content analysis, thematic analysis)
Relationship with participants		
6.	Relationship established	Contact was made by e-mail (MD) to arrange an appointment. The following topics were indicated in advance by e-mail: A. Reasons for participating in the online program "8 Lives"; B. Experiences with using the online program "8 Lives"; C. Changes after participation in the program; D. Evaluation of the "8 Lives" program; E. Consideration of different experiences with suicide or suicidality in the program.<br>Before the telephone interview began, there was an opportunity to clarify open questions and for small talk.
7.	Participant knowledge of the interviewer	The participants could have known that MD was part of the team that developed the online program as it was described on the website.
8.	Interviewer characteristics	Profession as a psychologist, affiliation to Department of Medical Psychology, University Medical Center Hamburg-Eppendorf, member of the project team
**Domain 2: study design**		
Theoretical framework		
9.	Methodological orientation and Theory	Structuring qualitative content analysis according to Kuckartz, U. (2018). Qualitative Content Analysis: Methods, Practice, Computer Support. 4th edition. [Qualitative Inhaltsanalyse: Methoden, Praxis, Computerunterstützung. 4. Auflage], following a realistic paradigm (Potter, J., & Wetherell, M. (1987). *Discourse and social psychology: beyond attitudes and behaviour*. Sage)<br>Please see methods section: ***Qualitative approach: Follow-up telephone interviews (page 9).***

No	Item	Description or section in the manuscript
Participant selection		
10.	Sampling	How were participants selected? *e.g. purposive, convenience, consecutive, snowball*<br>At the end of the post-assessment, study participants who completed the online program could optionally leave their contact details in an online input mask. Interested persons were contacted by e-mail approximately 12 weeks after completion of the online program and received further written study information about the telephone follow-up interview. If they were still interested in a telephone interview about the online program, they were requested to sign a consent form to participate in the follow-up interview. The send the consent form via mail. If this was returned, a telephone interview appointment was arranged; 12-26 weeks after program completion.
11.	Method of approach	email and telephone
12.	Sample size	16
13.	Non-participation	13 of 30 persons (43.3%) did not reply to invitation mail. One person answered not be interested in a telephone interview anymore without specifying further. See also ***page 18-19.***
Setting		
14.	Setting of data collection	Via telephone; participants were at home or in a clinic.
15.	Presence of non-participants	Presumably, no other person was present during the interview apart from the participant and the researcher. We had recommended an undisturbed place for the interview in advance. However, since we conducted a telephone interview, it cannot be ruled out that the interviewed persons were not alone – although it seemed so.
16.	Description of sample	Important characteristics (e.g. demographic data) of the sample are displayed in ***table 3*** in the manuscript (section results/qualitative approach).
Data collection		
17.	Interview guide	Please see ***S7 table*** for the semi-structured guide that was used. The interview guide developed following the guidance of *Helfferich, C. (2011). The quality of qualitative data [Die*

No	Item	Description or section in the manuscript
		Qualität qualitativer Daten] was pilot tested with another study team member.
18.	Repeat interviews	There were no repeat interviews carried out.
19.	Audio/visual recording	We used digital audio recording to collect the data.
20.	Field notes	There were no field notes made during and/or after the telephone interview. Handwritten notes were taken during the interview of what the interviewee said. On the one hand, to be able to better refer to what was said during the interview, and on the other hand, as a safety backup in case the audio recording did not work for technical reasons (see also ***S7 table***).
21.	Duration	The average duration of the telephone interviews was 30 minutes (SD=12) with a range from 11 to 59 minutes. Please see ***table 3.***
22.	Data saturation	N/A (No grounded theory approach was used)
23.	Transcripts returned	Transcripts were not returned to participants for comment and/or correction.
**Domain 3: analysis and findings**		
Data analysis		
24.	Number of data coders	2 (MD, JB)
25.	Description of the coding tree	Please see ***S8 table (coding tree)*** for a description of the coding tree as well as results in manuscript (***page 18-21***).
26.	Derivation of themes	Themes were identified in advance and derived from the data (deductive and inductive). Please see ***Qualitative approach: Follow-up telephone interviews (page 9).***
27.	Software	MAXQDA 18 and 20 (VERBI)
28.	Participant checking	Participants did not provide feedback on the findings.
Reporting		

No	Item	Description or section in the manuscript
29.	Quotations presented	Yes, participant quotations were presented to illustrate the themes / findings. See ***table 4a and 4b*** and ***S8 table (coding tree).***
30.	Data and findings consistent	Yes, we think that data and findings presented are consistent, but also think that it is the readers' and reviewers' right and duty to judge about that.
31.	Clarity of major themes	Yes, we think that major themes presented, but also think that it is the readers' and reviewers' right and duty to judge about that. See ***page 18-21,*** including ***table 4a and 4b.***
32.	Clarity of minor themes	Yes, we think that there is a description of diverse cases and discussion of minor themes but also think that it is the readers' and reviewers' right and duty to judge about that. See ***result section (pages 18-21),*** including ***table 4a and 4b, discussion (pages 24-28)*** and ***S8 table (coding tree).***

Note. Checklist by Tong, A., Sainsbury, P., & Craig, J. (2007). Consolidated criteria for reporting qualitative research (COREQ): a 32-item checklist for interviews and focus groups. *International journal for quality in health care*, *19*(6), 349-357.
