# Supplementary material for: Evaluation of an online suicide prevention program to improve suicide literacy and to reduce suicide stigma: A mixed methods study
Source: PLoS One. 2023 Apr 28;18(4):e0284944. doi: 10.1371/journal.pone.0284944 (PMC10146514; doi:10.1371/journal.pone.0284944)
Supplement: S5 Table — (PDF) [file pone.0284944.s005.pdf]

S5 Table. Good Reporting of A Mixed Methods Study (GRAMMS) checklist

Table S5. GRAMMS checklist (O'Cathain et al., 2008)

Guideline	Section: page
Describe the justification for using a mixed methods approach to the research question	Methods: pg. 11
Describe the design in terms of the purpose, priority and sequence of methods	Methods: pg. 11
Describe each method in terms of sampling, data collection and analysis	Methods: pg. 7-11
Describe where integration has occurred, how it has occurred and who has participated in it	Methods: pg. 11
Describe any limitation of one method associated with the present of the other method	Results: pg. 22-23
Describe any insights gained from mixing or integrating methods	Results: pg. 22-23; Discussion: pg. 24-28

Note. Checklist by O'Cathain, A., Murphy, E., & Nicholl, J. (2008). The quality of mixed methods studies in health services research. *Journal of health services research & policy*, *13*(2), 92-98.
