# Supplementary material for: Evaluation of an online suicide prevention program to improve suicide literacy and to reduce suicide stigma: A mixed methods study
Source: PLoS One. 2023 Apr 28;18(4):e0284944. doi: 10.1371/journal.pone.0284944 (PMC10146514; doi:10.1371/journal.pone.0284944)
Supplement: S6 Table — (PDF) [file pone.0284944.s006.pdf]

S6 Table. Exploratory subgroup analyses

Table S6a. Exploratory subgroup analyses – Global p-values of interaction term

	Primary Outcomes				Secondary Outcomes				
	**Suicide literacy**	**Perceived suicide stigma** ***SOSS-SF adapted***			**Self-stigma*** ***SOSS-SF adapted***			**Self-efficacy expectations**	
	LOSS-SF	***Stigma***	***Isolation/ Depression***	***Normalization/ Glorification***	***Stigma****	***Isolation/ Depression****	***Normalization/ Glorification****	***SWEP-6***	***SWEP-7****
**Online program variant**	**.01**	.28	.29	.39	.37	.84	.41	.60	.27
**Gender**	.71	.09	.34	.34	.79	.94	.50	**.001**	**<.001**
**Age**	.78	.46	**.01**	.31	**.03**	.16	.56	.85	.45
**Education**	.18	.61	**.03**	**.001**	.95	.83	.75	.38	.48
**Size of residence**	.08	.58	.45	.20	.67	.84	.19	.70	.38
**Distress (t_0)**	.84	.85	.69	.91	.19	**.02**	.62	.80	.65

Note. We predefined the following subgroups: online program variant (1-5), gender (female, male, diverse), age (18-29, 30-39, 40-49, 50-59, >60 years), level of education (lower education ≤11 years of education, higher education >11 years of education), size of residence (city, medium sized town, small town, rural community), and distress level at baseline (lower <5, higher ≥5). For exploratory detection of differences between subgroups across all primary and secondary outcomes, this table shows the global p-values based on the interaction term (completer only, total N=268, *total N=168). **White cells: Significant differences ($p < .05$) between subgroups based on interaction term. Grey cells: No significant differences between subgroups. Results should be interpreted with caution due to a possible inflation of Type I error. We did not adjust for multiple comparisons.** Covariates included age, gender, education, size of residence, variant of the online intervention, and distress. A total of 46 comparisons (85%) showed no significant outcome differences between subgroups. In eight comparisons (15%), we found significant differences between subgroups. Please see Appendix F2 for exploratory subgroup analyses pre-post differences.

Table S6b. Exploratory subgroup analyses – EMMs for completer only

		Primary Outcomes								Secondary Outcomes									
		Suicide literacy[1]		Perceived suicide stigma *SOSS-SF adapted*[2]						Self-stigma* *SOSS-SF adapted*[2]						Self-efficacy expectations[3]			
		LOSS-SF		*Stigma*		*Isolation/ Depression*		*Normalization/ Glorification*		*Stigma**		*Isolation/ Depression**		*Normalization/ Glorification**		*SWEP-6*		*SWEP-7**	
**Subgroup**	**Factor**	**EMM**	**95% CI**	**EMM**	**95% CI**	**EMM**	**95% CI**	**EMM**	**95% CI**	**EMM**	**95% CI**	**EMM**	**95% CI**	**EMM**	**95% CI**	**EMM**	**95% CI**	**EMM**	**95% CI**
-	**Overall (N=268)**	**1.85***	1.6; 2.1	.02	-.07; .12	-.02	-.11; .07	-.08	-.16; .01	-.09	-.12; .01	**-.14***	-.26; -.01	**-.11***	-.22; -.01	**.23***	.09; .37	**.25***	.06; .44
**Online program variant**	**1: Suicidal ideation** (n=102)	2.34	1.94; 2.74	-.04	-.19; .11	.03	-.12; .18	-.14	-.27; -.01	-.12	-.24; .00	-.13	-.29; .04	-.15	-.29; -.01	.12	-.12; .33	.16	-.09; .41
	**2: Suicide attempt** (n=66)	1.28	.79; 1.77	.02	-.16; .21	-.10	-.28; .08	-.12	-.28; .04	-.03	-.18; .11	-.15	-.36; .05	-.06	-.23; .11	.35	.08; .63	.38	.08; .68
	**3: Loss by suicide** (n=28)	2.04	1.28; 2.80	-.12	-.41; .17	.21	-.07; .49	-.02	-.27; .23							.24	-.19; .67		
	**4: Caring for suicidal person** (n=17)	1.88	.92; 2.84	.29	-.07; .65	-.02	-.37; .34	.15	-.16; .45							.48	-.06; 1.02		
	**5: Interested/Other** (n=55)	1.50	.93; 2.07	.15	-.07; .36	-.13	-.34; .08	.01	-.18; .19							.20	-.12; .52		
**Gender**	**Female** (n=202)	1.84	1.56; 2.13	-.03	-.14; .07	.02	-.09; .12	-.06	-.16; .03	-.10	-.21, .00	-.12	-.27; .04	-.08	-.20; .05	.37	.22; .53	.48	.26; .70
	**Male** (n=56)	1.94	1.39; 2.50	.23	.02; .44	-.15	-.35; -.05	-.06	-.24; .12	-.03	-.23; .17	-.16	-.43; .12	-.21	-.45; .02	-.22	-.52; .08	-.40	-.80; -.01
	**Diverse** (n=10)	1.33	.01; 2.68	.04	-.45; .53	.03	-.46; .51	-.39	-.82; .04	-.05	-.47; .37	-.22	.36; -.80;	-.25	-.74; .24	-.28	-1.00; .44	-.27	-1.00; .50

Factor		*LOSS-SF*		*Stigma*		*Isolation/ Depression*		*Normalization/ Glorification*		*Stigma**		*Isolation/ Depression**		*Normalization/ Glorification**		*SWEP-6*		*SWEP-7**	
**Age**	**18-29 years** (n=104)	2.03	1.6; 2.4	.03	-.12; .18	.05	-.10; .19	-.03	-.16; .10	-.03	-.18; .12	-.03	-.24 .18	-.17	-.35; .01	.29	.06; .52	.32	.01; .64
	**30-39 years** (n=49)	1.60	1.01; 2.18	-.12	-.34; .10	-.04	-.25; .17	-.25	-.43; -.06	-.11	-.31; .10	-.13	-.42; .16	-.11	-.35; .14	.22	-.11; .55	.50	.06; .93
	**40-49 years** (n=42)	1.68	1.05; 2.31	.05	-.19; .28	-.35	-.58; .13	-.10	-.30; .10	.13	-.08; .34	-.04	-.34; .25	-.08	-.33; .17	.08	-.27; .43	.06	-.50; .38
	**50-59 years** (n=57)	1.87	1.32; 2.42	.15	-.05; .35	.04	-.15; .24	.02	-.16; .19	-.28	-.47; -.08	-.29	-.57; -.02	.01	-.23; .24	.27	-.03; .58	.20	-.22; .60
	**60+ years** (n=16)	1.81	.80; 2.82	-.09	-.47; .28	.31	-.05; .67	-.09	-.41; .23	-.46	-.93; .01	-.79	-1.45; -.14	-.46	-1.02; .10	.07	-.48; .63	.05	-.94; 1.04
**Education**	**Lower** (≤11 YED) (n=73)	2.10	1.62; 2.57	.07	-.10; .24	.14	-.03; .31	-.24	-.39; -.09	-.07	-.22; .08	-.12	-.33; .10	-.14	-.33; .04	.13	-.13; .40	.16	-.16; .48
	**Higher** (>11 YED) (n=189)	1.72	1.42; 2.02	.02	-.09; .13	-.09	-.19; .02	.01	-.08; .10	-.08	-.19; .04	-.15	-.31; .02	-.11	-.24; .03	.27	.10; .44	.31	.06; .56
**Size of residence**	**City** (n=143)	1.56	1.23; 1.89	.04	-.09; .16	-.04	-.16; .09	-.10	-.21; .00	-.13	-.25; .00	-.18	-.36; -.01	-.09	-.23; .06	.28	.10; .47	.38	.12; .63
	**Medium sized town** (n=46)	2.15	1.56; 2.74	-.11	-.33; .11	-.01	-.22; .21	-.06	-.25; .13	.02	-.20; .23	-.03	-.32; .27	-.33	-.58; -.09	.16	-.17; .49	.12	-.32; .57
	**Small town** (n=34)	2.38	1.70; 3.07	.06	-.20; .31	.15	-.10; .40	.13	-.09; .35	-.03	-.27; .20	-.12	-.44; .21	.06	-.21; .33	.04	-.34; .43	-.08	-.55; .40
	**Rural community** (n=32)	2.12	1.42; 2.83	.12	-.14; .38	-.14	-.40; .12	-.19	-.41; .04	-.13	-.40; -.15	-.13	-.51; .26	-.13	-.45; .20	.25	-.14; .64	.29	-.29; .86
**Distress (t_0)**	**Lower (<5)** (n=66)	1.89	1.39; 2.39	.04	-.15; .23	-.05	-.23; .13	-.07	-.23; .09	-.23	-.47; .01	-.50	-.82; -.18	-.05	-.32; .22	.26	-.02; .54	.14	-.34; .63
	**Higher (≥5-10)** (n=202)	1.83	1.54; 2.12	.02	-.09; .13	-.01	-.11; .10	-.08	-.17; .02	-.06	-.14; .04	-.07	-1.00; .21	-.13	-.24; -.09	.22	.06; .38	.27	.06; .47

Note. Exploratory subgroup analyses (completer only, total N=268) by online program variants, sociodemographic characteristics and level of distress in primary and secondary outcomes. [1]= scale 0-12. Higher values indicate higher suicide literacy. [2]=scale 1-5. Higher values indicate higher stigma. [3]=scale 0-10. Higher values indicate higher self-efficacy expectations to be able to get support. YED= years of education. Covariates included age, gender, education, size of residence, variant of the online intervention, and distress. **White cells: Significant differences (p < .05) between subgroup outcomes based on interaction term. Grey cells: No significant differences between subgroups based on interaction term** (see appendix F1). Results should be interpreted with caution due to a possible inflation of Type I error (not adjusted for multiple comparisons). *Total N=168.
