# Supplementary material for: Evaluation of an online suicide prevention program to improve suicide literacy and to reduce suicide stigma: A mixed methods study
Source: PLoS One. 2023 Apr 28;18(4):e0284944. doi: 10.1371/journal.pone.0284944 (PMC10146514; doi:10.1371/journal.pone.0284944)
Supplement: S7 Table — (PDF) [file pone.0284944.s007.pdf]

S7 Table. Semi-structured guide for telephone interviews on online suicide prevention program “8 lives”

Table S7. Semi-structured guide developed for the online suicide prevention program

**Call introduction**	**Notes**
Hello Ms/Mrs/Mr ..., this is Mareike Dreier from the University Medical Center Hamburg-Eppendorf. We scheduled an appointment for the interview today. Is it convenient for you right now?<br>[Smalltalk]<br>Before we start with the interview, let me tell you something about the **background of the interviews**. We are conducting the interviews with you and other participants of the program "8 Lives – Experience reports and things to know about suicide". As you know, we developed the online program as part of the 4E project and would like to learn more about your experiences with the program. This allows us to further improve our program but also to gather suggestions for other scientists on what should generally be considered in such programs. Do you have any questions?<br>[answer questions]<br>During the interview, I will take **handwritten notes**. The interview will be recorded for later data analysis; the audio recording will be transcribed (excluding names or places mentioned by you) and deleted afterwards. Everything you tell me will be treated confidentially. The data will be analyzed anonymously, i.e., no conclusions can be drawn about your identity. It is possible that parts of the transcripts will be used for scientific publications. The interview will take about 20-40 minutes. Do you have any questions about the process in advance?<br>[answer questions]	Interview number: ___

<table>
<tr><td colspan="3">Then we can start now, and we will begin with the interview and the recording.

[Start audio recording]</td><td></td></tr>
<tr><th>Guiding questions/narrative request</th><th>Possible topics and inquiries</th><th>Check</th><th>Maintenance questions</th></tr>
<tr><td colspan="4">Topic A: Ways to participation in "8 Lives" / motives / prior knowledge / prior experience</td></tr>
<tr><td>I would like to ask you to talk about your experience with the online program "8 lives - Experience reports and things to know about suicide ". Interesting for me is what is important to you....

I. …maybe you begin with how you became aware of the program?

II. Why did you participate?

III. People come to the program with different levels of prior experience. I would be interested to know how it is in your case.</td><td>A1. Ways to participate in the programm „8 Lives“ / motives
– A1.1. Access to the program: How did you become aware of "8 Lives"?
– A1.2. Motives for participation: Why did you participate?
– A1.3. Personal experience with suicidality: In the "8 Lives" program, there were different paths through the program, so that participants were presented different information (e.g. information for those affected themselves or for relatives). What kind of experience with suicide or suicidality did you indicate? [Path 1: suicidal ideation; Path 2: suicide attempt; Path 3: loss due to suicide; Path 4: concern for loved one; Path 5: generally interested/other]
A2. Prior knowledge / prior experience
– What did you think about suicidality/handling stressful situations at the time?
– A2.1. Retrospective assessment of own knowledge
– A2.2. Retrospective assessment of own stigma
– A.2.3 Retrospective assessment of own self-efficacy expectations</td><td>□ A1 --
□ A1.1
□ A1.2
□ A1.3
TrackNo.: __

□ A2 --
□ A2.1
□ A2.2
□ A2.3</td><td>What happened next?

Could you describe this (in more detail)?

What about...?

People come to the program with different amounts of prior experience. I would be interested to know how it is in your case.

What kind of experience with suicide or suicidality did you report?</td></tr>
<tr><td colspan="4">Topic B: User Experience / during program use</td></tr>
<tr><td>Some time has passed since you participated in the “8 Lives“ program.</td><td>– B. User Experience
○ B1. Memories: What do you remember about the program? (/Which chapters/content do you remember?)
○ B1.2 Reasons to remember: Why is xy memorable to you?</td><td>□ B1
□ B1.2
□ B2</td><td>Could you describe this in more detail?

What chapters/content do you remember?</td></tr>
</table>

IV. ***What do you remember about the program?***<br><br>V. ***I would be interested to know how it was for you to go through the program? /*** *How was it for you to use the program?* ***How did you think/feel about it?***	○ **B2.: Description/Use of the program:** What was it like for you to use the program? There were different chapters; video reports by various people...<br>▪ *About how long ago did you complete the program?*<br>▪ *How many breaks did you take? Did you go through the program in one turn?*<br>▪ *What did you think/feel?*		*How was it to work through chapter ...? (/videos / postcards / worksheets)*<br>*What did you think/feel while doing this?*
**Topic C. Changes due to participation / after program use**			
I would be interested to know if the program had an impact on you in any way after you completed it. ***So:***<br><br>VI. ***How did you experience the time after using the program? Did anything change?***<br><br>VII. ***Do you have the feeling that the program has helped you?*** *If yes, why? If no, why not?*	C. Stigma, knowledge, self-efficacy expectations related to participation in "8 Lives"<br>Has anything changed for you as a result of participating in "8 Lives"? If yes, what?<br>– **C1. Knowledge change:** Did you learn anything new by participating in "8 Lives"? If yes: What?<br>– **C2. Change of attitude:** Has anything changed in your perspective (/assessment/attitude) of suicidality and suicide as a result of participating in "8 Lives"? If yes: What?<br>– **C3. Behavior modification:** Has anything changed in your behavior as a result of participating in "8 Lives"? If yes: What?<br>– **C4. Change in self-efficacy expectations:** Has participation in "8 Lives" changed your self-efficacy expectations, e.g. to consult appropriate help or to talk about something that bothers you?	□ C1<br>□ C2<br>□ C3<br>□ C4	*What about ... in detail?*<br><br>*How was it immediately after completing the program?*<br><br>*How was it a few weeks later?*
**Topic D. Evaluation of the program**			
We would like to improve the program. Therefore, I would be very interested in your opinion:	D. Retrospective evaluation of "8 Lives“<br>– **D1. Conclusion:** (With the benefit of hindsight...)<br>○ **D1.1 Helpful/less helpful:** What helped you most? What did not help at all?<br>○ **D1.2 Satisfaction/dissatisfaction:** What did you like? Why?	□ D1<br>---<br>□ D1.1<br>□ D1.2<br>□ D1.3	*Could you describe this in more detail?*

VIII. What did you find to be poorly implemented in the program? Why? *Disadvantages? Drawbacks? What helped the least or not at all? Why? What would you remove? Why? What would you add? Why?*<br><br>***IX. What is well implemented in the program? Why?/*** *What helped you the most in the program? Why? What was especially important to you? What did you find good? Why?* ***On which aspects would you like to see more content / information?***	○ **D1.3 Benefits**<br>○ **D1.4 Difficulties:** In your opinion, what are drawbacks/disadvantages of the program? What did you find poorly implemented?<br><br>– **D2. Ideas for improvement:**<br>○ **D2.1 Different structure:** Would you structure parts of the program differently? If yes, what?<br>○ **D2.2 Too much/too little/missing:** What would you remove? Why? What would you add? Why?	□ D1.4<br><br>□ D2<br>---<br>□ D2.1<br>□ D2.2	
**Topic E. Stigma experiences regarding suicidality: adequately addressed in program**			
Some people report being judged by others [for suicidality/suicide of a loved one], fearing being judged, or judging themselves.<br><br>***X. What experiences*** *(and/or evaluations)* ***are important to you in this context? Were such experiences*** *(and/or assessments)* ***sufficiently addressed in the program?***	E. Suicide stigma in the program: adequately addressed?<br>– **E1. Experienced stigma:** Have you experienced stigma because of your experience with suicidality? If yes: What kind of stigma?<br>○ E1.A. Has this been sufficiently addressed in the program?<br>– **E2. Perceived suicide stigma:** What are "typical" prejudices or stigmas regarding suicidality or suicide that you have encountered? (Or: that you think are present?).<br>○ E2.A. Have these been sufficiently addressed in the program?<br>– **E3. Self-stigma:** Have you noticed yourself judging yourself in a certain (/stigmatizing) way because of your experience with suicidality or suicide? If yes: How?<br>○ E3.A. Have these been sufficiently addressed in the program?	□E<br>---<br>□E1<br>□ E2<br>□ E3	*When you think about xy [stigma associated with suicidality/suicide], what was that like for you?*

Topic F. Conclusion			
You have gone through the entire program. What made you stick with it? *[Were there parts of the program/moments when you didn't want to continue working on it?]*<br><br>*Thank you - those were my questions about the program.*<br>*Could you think of anything else?* ***Is there anything left open that is important for you to mention?*** *What else is important for you to mention regarding the program? What has not yet been sufficiently addressed in the interview with regard to the program?*	**F. Possibility for further remarks**	□F	
**Topic G. Demographic questions**			
Finally, I would like to ask you 2 (/3) questions about yourself.<br><br>***Would you tell me how old you are?***<br><br>***(Which gender do you feel you belong to?)***<br><br>***What is your highest educational qualification?***	**G1. Age**<br>**G2. Gender**<br>**G3. Highest level of education**	□G1<br>□G2<br>□G3	
**[Farewell]** Then we have reached the end of the interview. I would like to thank you again very much for your openness and time! It was very helpful for us. If you think of anything else, please feel free to contact me.			

Note. The interview guide was developed by MD, supervised by SL and NP; following the guidance of Helfferich, C. (2011). The quality of qualitative data [Die Qualität qualitativer Daten]. The guide was developed and used in German and translated to English. Consideration of suicide risk: The interviewer explored whether the interviewed person was currently still having suicidal ideation,

how acute the suicidal ideation was, and whether the person has sufficient support in place. Additional to the guide displayed here, there was a structured emergency plan that was followed when a person was acutely suicidal.
