# Supplementary material for: Evaluation of an online suicide prevention program to improve suicide literacy and to reduce suicide stigma: A mixed methods study
Source: PLoS One. 2023 Apr 28;18(4):e0284944. doi: 10.1371/journal.pone.0284944 (PMC10146514; doi:10.1371/journal.pone.0284944)
Supplement: S8 Table — (PDF) [file pone.0284944.s008.pdf]

S8 Table. Coding tree

Table S8. Coding tree based on 16 follow-up telephone interviews

Main code	Subcodes and Description		Further explanation and/or example quotes
**Selected program variant**	1: **Suicide ideation**		Kind of lived suicide experience, i.e., program variant the participant worked on.
	2: **Suicide attempt**		
	3: **Loss by suicide**		
	4: **Caring for a suicidal person**		
	5: **Interested/Other**		
**Length of interview**			Code minutes in transcript.
**Demographics**	**Gender**		Female, Male.
	**Highest education level**		9 years of high school<br>10 years of high school<br>12-13 years of high school<br>University degree
	**Age**		Code age in years in transcript in years.
**Prior knowledge and prior experience regarding suicidality, suicide and suicide stigma**	**Professional context**	**Occupation**<br>Participants described having experience with suicidality as part of their professional work, mostly in health care with people with mental illness (e.g., as a psychological psychotherapist, counselor, social worker, nurse, researcher).	Background experience the participant has, e.g., being affected by suicidality (himself/herself or as a family member) – both in a professional (mainly program variant 5) or in a personal context (program variant 1-4).
		**Stigma**<br>Participants who work professionally in the field of suicide prevention reported a lot of shame regarding suicidality experienced by those affected. From participant's perspective, clients are often unprepared for situations, such as when they are admitted to a psychiatric ward. Clients often have difficulty explaining a suicide attempt. Participant reported that "stigmatization by others" was a new component for him/her although s/he had attended extensive seminars on suicidality as part of a professional training. Self-stigma and symptomatology were more at the focus of his/her work with patients with a mental illness.<br>From the perspective of the health care professionals, relatives who lost a close person by suicide often feel misunderstood, that they are shunned, and that friends and acquaintances show fear of contact and helplessness. This group often feels strong feelings	

		of guilt. Various prejudices are known ("only wants to be seen", "is not serious about suicidal thoughts", "wants to manipulate", "wants to blackmail"). From the perspective of a participant professionally working in this field: The younger a person who had suicided, the more critically the family was viewed from the outside, the more stigmatized they are.	
	**Private context**	**Own suicidal ideation/suicide attempt**<br>The range of lived experiences reported is very wide: Suicidal ideation, chronic suicidality over many years and suicide attempts. Some participants reported being affected by suicidality and suicides in several ways (also as a close person who lost a person by suicide). Some participants report having been in treatment for suicidal ideation currently or in the past.<br>Participants reported only disclosing suicidal ideation or a suicide attempt to certain people. Participants reported that persons are differently suited to confide in their own suicidal ideation/suicide attempts which depends on the degree of relationship with the person but also on persons' attitude to suicide. Participants reported to think carefully about who to disclose suicidal ideation to. Some participants report about smaller groups of people to whom they confide suicidal ideation or a suicide attempt (e.g., 1-3 close friends); in this small group "suicidality is taken seriously" and the participants stated to feel understood. Other participants also reported that suicidality could not be addressed anywhere except in psychotherapy or that suicidality had to be dealt with alone. Some reported experiences with therapists who would leave out the topic or only briefly tick it off because (from participants' point of view) therapist may be uncomfortable with suicidality.<br>Some of the participants report mental illness (e.g., schizophrenia, depression), which is experienced as stigmatized and partly concealed from others.	
		**Caring for close suicidal person**<br>Participants report kind of relationship to the person attempting to take his life and details on what has happened and how the suicide attempt affected the family.	
		**Loss by suicide**<br>The participants report different relationships to the suicidal person (e.g. brother, aunt, son) and time that had passed since the suicide.	
		**Stigma**	

		Program variant 1&2:<br>Potential stereotypes are known to most participants (e.g. too cowardly for life, weak, mentally unstable, should go to the loony bin). Some participants reported not having experienced any prejudices so far or being surprised by stereotypes presented in the program (e.g. cowardly); apart from surprise, no consequences are reported. Participants reported that they had not experienced any prejudice in their environment, but rather helplessness on the part of others; they had experienced that suicidal thoughts frighten other people, the topic of death anyway; people do not know what to do when confronted with the topic. This is described as very frustrating for other people and for oneself.	
**Way of access to the program**			How did the interviewee become aware of the program? Meant e.g. via advertisement on psychenet.de, internet search, reference on other website, poster, etc.<br>This is not intended to code lived experience of suicide.
**Motivations for participation**			Why did the person participate in the program? (Possibly overlaps with experience with suicidality); Here, only code what the decisive reason for participating in the program was, e.g., to get more tools professionally for suicidal clients; because one has/knows suicidal thoughts oneself and wants support, or similar.
**Experiences during program use**	Participants described a sense of general interest, curiosity, surprise, desire to learn more about the topic of suicidality, not being alone with the topic suicidality or suicide while using the program. Participants reported feeling compassion for people in video reports who described suffering and a feeling of being more detached from own issues. Participants, especially those from professional contexts, described that by working with suicidality on a daily basis, no distress had arisen during program use.<br><br>Participants also described that it was exhausting to reflect about suicidality/suicide and keep everything in mind. Participants described a mix of emotions; on the one hand the need to talk about suicidality, on the other hand feelings of guilt towards others.<br><br>Participants, mainly who have suicidal thoughts or made a suicide attempt, describe that during the use of the program the videos were emotionally distressing. Some participants reported that they found the videos helpful despite being distressing, while others reported that they skipped some videos because of distress.<br>Participants who lost a person by suicide reported they had been sad at some points during the program (“when I think of the person who died, I always get sad. That's part of it, to grieve, it's not that bad. Then that feeling has passed again.”), e.g., during the digital		**What was felt while using the program?**<br>Also, code here possible distress/unwanted side effect/undesirable events <u>during</u> program that do not constitute adverse events and are not permanent, i.e., do not still play a role after program completion. Examples:<br>-describing a temporary sad feeling because of thinking of a close person lost by suicide (especially program variant 3) or<br>-a description that the program is exhausting/distressing because of lived experience video reports but not for a longer period and to a minor extent (especially program variant 1+2), or<br>-being emotionally activated /affected by video reports of suicidal crises or video reports on loss by suicide (especially program variant 5).<br><br>**What was thought while using the program?**<br>E.g., “During the program use I thought the program was interesting/helpful”; “I thought I have similar experiences”, “I thought I need a break and will continue later.”

	postcard messages of the other participants. However, this sadness was explicitly described as not being distressing. Another participant describes a "tension" during use and reports a "certain openness to walk the path of mourning" is necessary when using the program. Feelings also came up that had not yet been processed, yet the participant stayed with the program and described the program overall as not emotionally upsetting.<br><br>One participant described perceiving a deterioration in her mood during use, emphasizing that many other influences may have contributed at the time of program use and that she did not causally attribute the deterioration to the program.<br><br>The program was described as intense during use. The program was described as an additional burden, with the participant adding "That something temporary is burdensome does not mean that it cannot be permanently good."		"Okay, I thought at the beginning, first of all, yes, super cool, see what it helps me and also how I can best support the establishment of such a program. I was still very open at that point in the beginning. And I already felt tense, a little bit, because approaching the topic [suicide], you also have to have a certain openness, also simply to walk the path of mourning, because every time you deal with the topic, of course the feelings that have not yet been processed come up again. That's why I was a bit tense at the beginning and that it really was. So what was I thinking? I thought it was good that it was divided into chapters, so I also took advantage of the fact that I had paused once in a while, and then I went straight through some of them and found it (.) well structured, so really as far as the subject matter is concerned, yes, that really does remain in the memory, as far as the subject matter chapters are concerned, so a little bit all around, and I also found it really very interesting." I6, participant lost a close person by suicide, 07:05min<br><br>"In the beginning I thought for a long time, is this good, what I am doing here? Because the program actually, I had the feeling that it was an additional burden to deal with the topic, so that I struggled through it for quite a while until I came to the coping strategies. (...) Last year I fought my way through so much literature and it was often so stressful. I somehow have the feeling that it's not so bad if something is burdensome, if it brings me further. So that something is temporarily burdensome doesn't mean that it can't be permanently good, so I wanted to do that too, burden or not. (...) I could somehow empathize with the individual stories from videos with people quite well, ultimately I didn't know it like that, how it must feel, so the feeling was partly a great consternation, on the one hand towards the reports, but also towards my own situation." I4, participant with suicidal ideation, 06:31min
**Evaluation and achievement of program objectives**	**Increase in suicide literacy**<br>Participants reported a deepening of knowledge, and a refreshing and repetition of what they have already known about suicidality and suicides. This refers to a general factual knowledge. Participants also reported an		Increase in suicide literacy:<br>-When participants indicated that pure factual knowledge increased or there was a refreshment, confirmation of existing knowledge or consolidation of existing knowledge.<br>-Increase in suicide literacy is also coded when participants describe that they experienced relief from the increase in knowledge about help seeking possibilities

	increase on a more personal level, e.g. a better understanding of own suicide attempts, an increase in personal coping skills, including knowledge about help options, understanding the need to get help, and feeling a permission to seek support.<br>A decrease in suicide literacy was not reported and therefore not coded. No change in suicide literacy was not reported and therefore not coded.		"ah, that's difficult to say, so overall the program, dealing more with this topic, seeing that you are not alone and have such problems, seeing that there are also solutions for it and that you just really have to address it [speak about suicidality] and also can and may, so that you get help. All in all, that was very, very helpful." I12, participant with suicidal ideation, 14:07min<br><br>This coding also applies when participants describe being better able to comprehend and categorize their own suicide attempt or suicidal ideation because of the information they received in the program.<br><br>"[After using the program] I have much more understanding for people who try to take their live. I can understand better why they do it and I can also understand better why I tried to do it myself. So I also have more understanding of myself. (…) This helped me to reflect, to think and it has helped to have more compassion, compassion for others, for whatever reason they try to take their live." I13, participant who survived a suicide attempt, 12:39min
	**Change in suicide stigma**<br><br>Participants reported prejudices they identified through participation in the program, a reflection and a subtle attitude change primarily through increased knowledge. Participants also described an increase in awareness of suicide stigma, even if they did not perceive any change in their own attitudes. Participants affected by suicidality reported relief at realizing they were not alone in dealing with the issue of suicidality and that the program had helped somewhat in breaking the silence on the topic. An	**No change**	Under the code "Change in suicide stigma", it is coded if participants state that they are more aware of the topic of suicidality, suicide and stigma - but no significant increase or decrease is described. It can also be coded here if people report having talked more about suicidality and stigma with others after the program, e.g. without being affected themselves.<br><br>"No change" is coded when participants explicitly state that the program had no impact, e.g., because they already had a fixed opinion on the topic suicidality.<br><br>"It didn't really change my attitude to the subject I would say, but rather confirmed or refreshed things." I15, participant lost a close person by suicide, 08:40min
		**Decrease in suicide stigma**	If individuals reported having more compassion and understanding for others who are affected, this will be coded as decrease in suicide stigma.<br>„[After using the program] it changed in a way that I don't feel like that anymore, yes, that I know that others feel the same way and that it [suicidality] is talked about or was talked about in the program. It was somehow an open way of dealing and that did me

	increase in stigma was not reported and therefore not coded.		good, also the time afterwards. This 'No, we don't talk about it', I think that's really bad because then I can't get rid of my [suicidal] thoughts. [The program] was quite a relief." I14, suicide attempt survivor, 09:38min
	**Change in self-efficacy expectations in dealing with psychologically difficult situations**	**No change**	"No change" was coded when participants state that they have already showed a particular behavior in difficult situations and therefore did not notice a higher self-efficacy expectations in dealing with difficult situations after completing the program.<br><br>"I think that [confidence in myself to seek help or to talk about something stressful with someone] I had already done before. I've been in therapy for one and a half years and long before the study, sometime last year in autumn (…)" I4, participant with suicidal ideation, 14:13min
		**Increase in self-efficacy expectations in dealing with difficult situations**	Participants stated that they had more confidence in being able to talk to others about psychological problems and suicidal thoughts, or that they generally had more confidence in telling others how they really felt after taking part in the program. One participant explained, "Because you see in the program that people dare to talk about it, and yes, very subtly then probably, and then I also dare to open up more to this topic; I found that very good. A decrease in self-efficacy expectations was not reported and therefore not coded.<br><br>"Well, I think that [through participation in 8 lives] I am now even more open [to seek help], that even if I were affected and somehow, I don't know, if I were to reach a point myself, for example, that I would tell others I don't want to live anymore or that I would have the courage to say so." I8, participant worried about a close suicidal person, 22:03min<br><br>"Because you see in the program that people dare to talk about it, and yes, very subtly then probably, and then I also dare to open up more to this topic; I found that very good." I6, participant lost a close person by suicide, 19:24min
	**Other changes after program completion**<br>After program completion, participants described	**Enhancement of the coping possibilities with one's own suicidality**	**Change in actual action:**<br>Participants who had suicidal thoughts or were suicide attempt survivors described **change in actual action,** e.g., talking about distress, disclosing suicidal thoughts, or seeking professional

	different changes which they attributed to program participation.		support (e.g., psychiatric clinic) as actual behavior change after the program completion. Some described that there was a change in behavior, but that they did not attribute it causally, or not exclusively, to program participation.<br><br>**Talked about distress**<br>"[What has stuck with me from the program] is that I now say something when I'm not feeling well and don't cover it up. That also took strength last year, always smiling and yes, and that I now say specifically what's going on." I1, participant who survived a suicide attempt, 11:02min<br><br>**Disclosed suicidal thoughts**<br>"[8 lives] actually encouraged me to talk about it [my problems]. So I've had phases like that for years, every now and then, and I've never talked about it with anyone before. So, neither with my family, nor with my husband. And this is the first time, and in principle it was actually through this program that I found the courage to talk about it." I12, participant with suicidal ideation, 08:41 min<br><br>**Sought professional support (e.g. going to a psychiatric clinic)**<br>"(...) It was also because of the program that I initiated these things [admission to a psychiatric hospital], because it was clear that action had to be taken, also for our family, yes. (...) [The program] has played a role in this respect, because it has set things in motion. It made it very clear that something has to happen now, because otherwise we will all slip further and yes, that I also have to change something if I want to stay alive or if I don't want to expose my children to the trauma of losing me." I5, participant with suicidal ideation, 14:10 min
			**Change in intended action**<br>Participants described **change in intended action**, e.g. wanting to take more care of oneself (e.g., in the sense of wanting to seek help, wanting to implement more positive activities in everyday life, or wanting to take more responsibility for own well-being).<br>"For me there were also some suggestions in the program about how to take care of yourself, mindfulness or what is good for you, that you should do that and as prevention. So, I'm trying to work on that more, so I thought about it afterwards, I thought about it again, what do you like to do? What else would you like to do in your free

			time?" I8, participant worried about a close suicidal person, 19:01 min
			**Change in the way you look at yourself**<br>Participants described **changes in the way they look at themselves** and attributed that to program participation. Participants described feeling more compassion for themselves, viewing their own lives as more valuable, feeling more hopeful, or feeling encouraged to continue living. Participants indicated that the program strengthened their ability to distance themselves from suicidal thoughts, have a better understanding of their own suicide attempt or thoughts (note: overlap with code increase in suicide literacy).<br><br>a) More compassion for oneself<br>b) Seeing one's own life as more valuable<br>c) Feeling more hope / feel encouraged to continue living<br>d) Distancing ability from suicidal thoughts strengthened<br>e) Better understanding of own suicide attempt or own suicidal thoughts (Note: overlap with subcode *increase in suicide literacy*)<br><br>"[After participating in the program] I paid more attention to my life, it was worth more to me afterwards. (...) [The program helped] to deal better with the fact that I attempted suicide and to deal better with my future life." I7, participant who survived a suicide attempt, 06:51min<br><br>"(...) there were two women [in the videos] with whom I could go along very well, one of them also conveyed quite well her perspective, the hope that was behind it. I was simply emotionally involved. It has also given me courage." I5, participant with suicidal ideation, 17:27min<br><br>(...) anyway, I found that this program is not an impulse for the people who want to kill themselves, it is a positive program, all the way through. I13, participant who survived a suicide attempt, 17:44min
		**Mood change**	**Deterioration of mood**

		One participant reported a deterioration in mood for several days, although she did not attribute this causally to program exposure. Some participants described that they felt better after program use or that the program was a relief.	"I try to remember because that was also a very difficult phase, I didn't feel so good afterwards. So, it's not just because of that (the program), but because of this preoccupation or because of the statement about what I know from the video or something, (…). Maybe it was more of a negative mood, you could say. (...) [The bad mood was] a two or three-week story. (...) It is always difficult to establish causality because there are many other influences. (...) this extremely emotional thing or something, like the motto, I can't deal with it constructively for myself, that this is better now." I10, participant with suicidal ideation, 15:27min
			**Improvement of mood** (e.g. experience of relief after program use)<br><br>"[The program] did me good [afterwards]." I8, participant worried about a close suicidal person, 9:38min<br><br>"(…) because I found out again that others feel the same way, that others also have such thoughts and that I am not so alone with them. That did me good and the [program] also helped me afterwards [after the program was completed]." I14, participant who survived a suicide attempt, 10:57min
		**Change in interaction with others on the topic of suicidality/stigma**<br>Participants described that after participation, they exchanged more with others about the issue of suicidality and stigma in general without being affected oneself. Participants with general interest in der topic described feeling more compassion and having more understanding for persons who are suicidal. Participants described they felt more confident in dealing with a suicidal person or a person who lost a close person by suicide and felt a reduction of insecurities. After participating in the program, participants describe the intention to deal more sensitively or openly with people who are suicidal (e.g. clients) or if a person tells them about the suicide of a close person. Participants report also actually being more sensitive for the topic after program participation.	-Talking more with others about the issue of suicidality and stigma in general without being affected oneself (overlap to code *change in stigma*)<br>-Feeling more compassion and having more understanding for others who are affected (overlap to code *change in stigma*)<br>-Feeling more confident in dealing with the issue (overlap to code *change in suicide literacy* and *potential mechanisms of action> Reduction of insecurities through knowledge transfer/education)*<br><br>"[After participating in the program] I (...) simply discussed it a bit more with other people. I found that quite good, because before that I really hadn't. I think people tend to talk about it in a negative way, "oh, have you heard?", I don't know, "someone somewhere has thrown themselves in front of the train again", and then I found it quite interesting to talk about it with some friends, I'll say about the pressure of suffering that people have or what the movement is about it, exactly. Just to think about it in a more reflective way and to talk to people about it." I11, participant had general interest in the topic, 06:09min

		**Explicitly negated other changes** after program use, e.g., program had no impact on personal reasons for staying alive, no impact on the individual grieving process.<br>One participant who had attempted suicide stated that while she had dealt with suicidality and the experiences of others in the program, ultimately everyone had to find their own reasons for staying alive and the program had no impact on reasons for staying alive. One participant reported that the program had had no impact on the individual grieving process. One participant stated that she herself was too involved in the topic for professional reasons and therefore nothing had changed.	"(...) [after participating in the program] rather less [changed], um (...), what do I put that down to? (..) rather less (...) yes, because the most important thing for me was that (..) or the most difficult thing was always, is, was always the emotional handling of it [the death by suicide]. The program didn't help me so much in that respect, which means that at the time when I did it, it didn't really help me. It was rather less valuable for me personally in that I can say that it has now brought me a big step forward, that I can say that it has really had a big influence on my life or on my path of mourning, where I can say, okay, I have now really taken a big step forward. (...) I would have liked to have had [the program] earlier, I would have liked to have come across it earlier." I6, participant lost a close person by suicide, 10:02min
		**Unspecific changes**	a) Unspecific "has helped further"<br>b) Program has "made more thoughtful for a while"<br>c) Program initiated reflection processes, without specifying this further
	**Overall evaluation**	**Positively highlighted**<br>– Overall<br>– Videos<br>– Information texts<br>– Interactive opportunities<br>Digital postcard messages<br>– Copings strategies<br>– Break opportunities<br><br>**Videos**<br>The participants positively emphasized the lived experience video reports. The videos were helpful because of the personal reference to the topic suicidality/suicide that was created in this way. Participant reported that one could see that others had managed to go on living and what had helped them. The reports conveyed hope to go on living, promoted empathy and made mental states of crisis more comprehensible.<br>The high heterogeneity of persons in video reports provided different ways of accessing the topic suicidality, was evaluated positively. Through the videos, one could see how to talk about suicidality or a suicide, one noticed that one was not alone with the topic of suicidality or loss through suicide. Participants report that they perceived	Good, positive, helpful, sufficiently dealt with, satisfied, benefited, exciting, interesting. Assign code if something was rather helpful. Examples: "I enjoyed the videos.", "I learned something from the videos."<br><br>"I found it quite good how the program is structured and that you progress from chapter to chapter. Especially with these breaks in between, which were quite good for me. It was also good for me to read that in principle many others feel exactly the same as I do and that there were actually people who were brave enough to have small videos made of themselves and talk about this topic [suicidality]." I12, participant with suicidal ideation, 04:54min<br><br>"One was not only a listener, but was also actively involved in certain parts (…)" I2, participant had general interest in the topic, 24:09min<br><br>"In general, I think it was also attractively designed, so having videos in it is always very good in principle, if it's not just dry facts in black and white." I9, participant had general interest in the topic, 09:51min<br><br>"(…) and then there was another woman, oh dear (.), who keeps having suicidal thoughts. She has them again and again. And that

		the videos as honest, partly intimate reports. Participants emphasized positively the concrete coping options reported by persons with a lived experience of suicide in the videos. The openness in the videos was perceived as courageous. Some participants also positively recall individual sentences or messages from videos. The length of the interview excerpts (1-4 minutes) presented in the videos was described as positive.<br><br>**Information texts**<br>The information texts on suicidality and the overall benevolent, friendly, empathetic and praising tone in the texts were positively highlighted.<br>Break opportunities<br>Participants positively highlighted the opportunities for breaks.<br><br>**Interactive opportunities** *and* **Digital postcard messages**<br>The general program structure in different chapters was remembered positively, as it gave the possibility of structuring as well as the alternation between videos, texts, own statements (e.g., possibility to anonymously share experiences); thus reflection was initiated (one was not only a recipient or consumer).	happened to me, yes, it happens to me too and somehow it's nice, it's not nice, but it's good when someone else has it too and you don't think alone, only I have something like that and I think like that and maybe I'm disturbed or something." I14, participant who survived a suicide attempt, 04:14min<br><br>"In my own family I notice how difficult it is to talk about it, but I have also made the experience that it is not good if you don't talk about it. That's what I thought was important about the program, that made it clearer. All those who were in the [lived experience video reports] talked about it." I5, participant with suicidal ideation, 21:35min
		**Negatively highlighted**<br>**Videos**<br>Participants report that it can be exhausting to watch the videos and therefore there is a desire to use an additional text option to set their own pace. One participant noted a statement in a video that did not help her, although she said it will not influence her. Participants report that they were able to be more compliant with some of the videos.<br>**Length**<br>Participants found the program too long and too much information, while others would have liked more underpinning information.	Bad, negative, unhelpful, dissatisfied; not benefited, harmful, burdensome. Assign code if something was rather unhelpful. Examples: "The texts were too long and unbalanced." "The videos were too burdensome for me."<br><br>"For me [it was] partly very, very difficult to watch these videos. I haven't watched all of them yet. For me, it would have been better to have the text, to be able to read what they're saying, what they're expressing, because when I'm reading, I can decide for myself at what pace I'm going to proceed, or whether I'm going to end after a sentence or take a break, instead of watching the videos, which was sometimes very difficult for me."<br>I12, participant with suicidal ideation, 04:54min
		**Neutral**<br>Participants' statements cannot be assigned to a positive or negative evaluation, e.g. that participants cannot say anything about the question or that participants	The program element is mentioned, but it is not clear whether it was considered good or bad, helpful or not helpful etc. (e.g. "There were videos.")

<table>
<tr><td></td><td></td><td>emphasize that they do not give a positive or negative evaluation in this respect. For example, that there are videos.</td><td>“But that is nothing negative or not a minus point. If I had to write my own experiences, opinions (…). That was just what I couldn't do. I actually didn't do that at all in the beginning in the first rounds. At the end, I think, I wrote something. But that is certainly due to my little or no experience with the topic. I just didn't have anything that came to mind that I could write about it.” I2, participant had general interest in the topic, 10:50min</td></tr>
<tr><td rowspan="3">Feedback and ideas for improvement</td><td colspan="2">Extent
Note: Here only ideas for improvement are displayed.
We identified following ideas of participants:
-Videos should not be longer than 3 minutes
- Participants had different ideas about whether chapters should be unlocked successively. All chapters could only be accessed after the program was finished. While some participants found successive unlocking structuring and helpful, others would have preferred the option to move more freely through the program. One participant pointed out that important basic information might be skipped if this was possible. One participant suggested that past chapters could be accessed directly. One participant suggested that depending on the target group (be affected or professional) or preference of a person it could be differently.</td><td>Was the scope of the overall program and its individual elements appropriate? Should it have been longer/shorter?</td></tr>
<tr><td colspan="2">Layout/Design
No improvement reported.</td><td>Was the design/layout appealing and "catchy" or could/should it be better/different?</td></tr>
<tr><td colspan="2">Content
Note: Here only ideas for improvement are displayed.

Participant with suicidal ideation (program variant 1):
Videos also as text option.
Being able to control the degree of confrontation with the topic is important (see overall evaluation > negatively highlighted)

Participants who survived a suicide attempt (program variant 2):
Even broader group of persons in lived video experience reports, e.g. younger persons (15-18 years), persons from all different social backgrounds

Participants who lost a close person by suicide (program variant 3):
One participant would like more information on coping with grief and guilt. From his perspective, what remains after a suicide is often the big question “why”. Another participant stated that references to local self-help groups were included but could be emphasized more.</td><td>Content-related things in the program are meant, which could be supplemented, expanded, or presented in a shorter way, for example. What content was too much/too little in the program and could have been reduced/added?</td></tr>
</table>

	Professionals/interested participants (program variant 5):<br>Desire for the presentation within the program of<br>-conversation techniques with suicidal persons<br>-clear indications to recognize how acute suicidality is<br>-screening sheets to assess suicide risk which can be used with clients<br>-consideration of underage children in a family with a relative being suicidal/a relative died by suicide. Explanation what needs to be taken into account.<br><br>***Overall:***<br>-Paying more attention to different cultural backgrounds, different religions, etc.<br>-An interview with all persons from the lived video experience reports together. Interview questions on how the families deal with the fact that they speak publicly about the experience within the program, and how the families deal with the lived experience of suicide in general. Pointing out differences and similarities between the lived experience team members. Elaborate in a video together on the different ways of dealing with disclosure of the lived experience of suicide and how the differences (non-disclosure for a longer time, disclosure to certain family members, …) has then affected the person.<br>-Resource activation also in between the chapters, not as a fixed block.<br>-Personal output received at the end of the program based on everything in the program that was relevant for the person or what the person wrote within the program.<br>-Idea to include book recommendations.	
	**Technical implementation**<br>*See difficulties one line below.*	What could be technically better implemented?
	**Difficulties**<br>Participants reported having worked on chapters several times because they did not get further in the program for technical reasons.	Where were difficulties (technical or content) in the program? [Possible overlap with Adverse Events] This is about the time during the use of the program, not about consequences. Problems in content are meant, e.g., comprehension problems. Or difficulties in the program.
	**Composition**<br>*Note: Here only ideas for improvement are displayed.*<br>-Access to coping strategies should be earlier in the program.	What should be structured (fundamentally) differently in the program? Should jumping between chapters be possible? Login should be omitted?
**Adverse Events**	*Note: Adverse events could be: Suicidality increases; feeling more ashamed, etc.*	
	**Reports any adverse event**<br>Interviewed participants did not report adverse events. Reported was a deterioration in mood from participant. The participant did not link this to the program use. We coded as "other changes".	The interviewee directly states that this was a side effect/undesired effect of the 8 Lives program that he/she noticed, e.g., increase of suicidal thoughts.<br><br>The interviewee does not directly link a possible negative effect/side effect/adverse event to the program themselves. Raters code also if there is possibly a connection with what is reported and the program. Thus, strongly contains an

		interpretation by the rater and therefore may be somewhat "between the lines".
	**Actively negates an adverse event**<br>Interviewed participants negated actively adverse events.	The interviewee has denied questions about adverse events or has a theory/idea as to why they experienced something negative/adverse and does not relate it to the 8 Lives program.<br><br>"(…) anyway, I found that this program is not an impulse for the people who want to kill themselves, it is a positive program, all the way through." I13, participant who survived a suicide attempt, 17:44min
**Potential mechanisms of action**	*Note: Objectives of the program: to reduce the taboo surrounding suicidality (and then to speak openly with confidants); to get help in crises; to destigmatize (=reduce feelings of guilt and shame regarding suicidality); to increase the expectation of self-efficacy in dealing with psychologically stressful situations; to increase knowledge. The aim of the program is not to normalize suicides, but to classify them without condemning those affected or close persons of those affected,*	
	**Handing out of coping strategies and concrete instructions for action given**<br>Participants described coping strategies as helpful; especially also ideas how to talk about suicidality with health care professionals. Also, the pure fact that it is important to reach out to professionals and the relief to get "the allowance" to get help is reported.	Strategies in each of the areas of behavior, body, thoughts, and feelings was given. Could lead to the increase of action competence. E.g., texts explained how to talk to a doctor. Or case studies were presented.<br><br>"For example, these very unpleasant thoughts about myself, I knew beforehand that this was not good, but I had no starting point, I always had the feeling that I didn't like this self-optimization stuff of thinking positively (laughs lightly), but to ask myself, is this a helpful thought [as presented in chapter 5], I was able to start something with that. From the experience reports, I could relate to some of the things and I'm also sure that I'll watch the videos again in a week or two or sometime in between, when I happen to be sitting here anyway." I4, participant with suicidal ideation, 10:51min<br><br>"ah, that's difficult to say, so overall the program, dealing more with this topic, seeing that you are not alone and have such problems, seeing that there are also solutions for it and that you just really have to address it [speak about suicidality] and also can and may, so that you get help. All in all, that was very, very helpful." I12, participant with suicidal ideation, 14:07min
	**Not being alone with the topic suicidality/suicide; feeling of belongingness**<br>Participants from program variants 1 (suicidal ideation) and 2 (suicide attempt) described they do not feel so alone with the issue suicidality. They described this as positive effect of program participation.	"And women and men in different age groups and (.) I would say that was already a special experience for me (exhales) when I read, heard about the life experiences of other people and other countries. It gave me the feeling that I am not the only one with

		my problems. I knew that anyway, but it is a nice feeling to hear people who also have all kinds of psychological problems, yes." I13, participant who survived a suicide attempt, 08:15min<br><br>"Yes [the program was helpful] because I found out again that others are also like that, that others also have such thoughts and that you are not so alone with them. That did me good and it also helped me afterwards." I14, participant who survived a suicide attempt, 10:57min
	**Change in guilt and shame**<br>One participant explains that it was a good feeling to read messages from others. It could be interpreted as a small change in shame experience, although this is highly interpretive by the coders.<br>Another participant described no direct change in guilt, but a change in action despite guilt.	Possible overlap with subcode change in stigma.<br><br>"It's such a sense of shame that emanates from [my family]. For me, it was nice to read about it [suicidality/suicide] as well as other people did [within the program]. I also liked the different notes from all the participants [digital postcard messages] at the end [of the program] about what everyone had experienced. I don't know, it was kind of nice to see that it is somehow a topic where you have to talk about it and which also heals when you talk about it." I8, caring for a close suicidal person, 9:38min<br><br>"On the emotional level, it was such a mixed bag, so on the one hand it [the program] gave me the stimulus to talk about how I'm doing, that I'm not doing well. But at the same time, I also had feelings of guilt that by talking about it [suicidality], I was causing my children to worry and fear. That is simply also an issue and it still preoccupies me. I feel guilty that they [family members] are not well either, yes, I don't know, there is simply a conflict that could not be solved, because if one should talk about it and I think it was important that I talk about it, and it was also the case that my [adult daughter] had to take me to the hospital because I was acutely suicidal, that she also understands that, yes. But it is still extremely stressful and, yes, for me it has been important, the program, to also understand, yes. That it's just that you can't really do anything about it, you can't really influence the emotions of others. I can only hope that by going to therapy I am showing them that I care." I5, participant with suicidal ideation, 11.40min
	**Conveying hope**<br>Participants explained that the program conveyed hope. See also code overall evaluation > positively highlighted and other changes.	Protagonists (=persons with a lived experience of suicide in the video reports) convey hope for a way out.<br>"(…) anyway, I found that this program is not an impulse for the people who want to kill themselves, it is a positive program, all

		the way through." I13, participant who survived a suicide attempt, 17:44min<br><br>"(...) there were two women [in the videos] with whom I could go along very well, one of them also conveyed quite well her perspective, the hope that was behind it. I was simply emotionally involved. It has also given me courage." I5, participant with suicidal ideation, 17:27min
	**Protagonists as "role models"**<br>Participants reported that the simple fact that persons in the lived experience video reports talked about suicidality or a suicide (disclosure) this helped to see how to talk about suicidality and that talking about it is possible at all.	Protagonists show how to talk about suicidality.
	**Contact/Encounter with authentic persons who actually experienced suicide/suicidality**<br>Participants reported that the interviews were honest and credible.	Protagonists report honestly about their experiences, credibility of what is said is very high, because the protagonists are no actors. Protagonists are perceived as authentic.<br><br>"You can just sense something like that when a person speaks honestly, tells something honestly. That made me stay with it [the program]. So, the focus for me was not on how dramatic the story was, but that it was honest, that the person was honest in what s/he told. That touched me particularly." I13, participant who survived a suicide attempt, 27:42min
	**Identification possible because of heterogeneous protagonists**<br>Participants reported that different perspectives of different person on the topic suicidality were helpful, even though some lived experience reports were reported as more helpful than others. A participant said you could "pick out the videos" you found helpful. Some videos you could not agree with without specifying this further.	Since different people (age, gender, affectedness) were interviewed, one had different people with whom one could identify more or less. "Something for everyone" + diversity of the topic presented
	**High degree of openness**<br>Participants reported that they were impressed about the openness of the lived experience video reports and that seeing others talking openly about their experiences helped. (See also protagonists as "role models").	Protagonists talk openly about their experiences<br>"(…) I felt addressed by the people who participated (…) and by their inner openness to talk about what had been hurt." I13, participant who survived a suicide attempt, 27:42min
	**Possibilities for interaction/personal narrative sharing**<br>Participants pointed out that one was not a mere listener and experienced that as a helpful element that prompted self-reflection on the topic.	Participants can get involved in the program, e.g. by answering questions, filling out worksheets, leaving postcard messages to share their personal story/experience.
	**Reduction of insecurities through knowledge transfer/education**<br>Participants, especially in program variant 5 (interested) pointed out that they feel safer in interacting with persons having a lived suicide experience. The knowledge increased by videos and texts.	Knowledge often provides security. Stigma can make a person less likely to open up. If a person knows more about a taboo topic, they may be more likely to talk about it and seek help if they are in a suicidal crisis themselves or are concerned about a close one.

	**Stimulation of self-reflection**<br>See code >Possibilities for interaction/personal narrative sharing	Program stimulates self-reflection.<br><br>"(...) because it [the program] is so diverse and so comprehensive, it is impossible to sum it all up in one place. I think that the program is definitely suitable for dealing with it [suicidality], which was also the case for me, in order to create clarity. I found the myths important to break down. And because it made me think again about where it [suicidal thoughts] occurred in my case, in my environment, how others deal with it, how I deal with it myself." I5, participant with suicidal ideation, 25:40min
	**Promoting empathy**<br>Participants reported that they felt more empathy on an emotional but also on a cognitive level (more understanding). This was explained by the lived video experience reports.	Protagonists promote empathy.<br><br>"Now I can understand a little bit the motives behind it, or the pressure of suffering behind it." I11, participant had general interest in the topic, 08:19min<br><br>"[After using the program] I have much more understanding for people who try to take their live. I can understand better why they do it and I can also understand better why I tried to do it myself. So I also have more understanding of myself. (…) This helped me to reflect, to think and it has helped to have more compassion, compassion for others, for whatever reason they try to take their live." I13, participant who survived a suicide attempt, 12:39min
	**Transparency**<br>We found no statements for this predefined subcode.	There is transparency about who is involved, and how, in the project.
	**Participative program development**<br>Participant stated that she liked the "trialogical approach" (experts by experience, experts by education and relatives of those affected) because she in general thinks that this is the best way to deal with such topics. Another participant was interested in the involvement of the lived experience team and appreciated it.	Person with lived experience were involved in development process.
	**Autonomy while using the program**<br>-Participant described she liked break opportunities; therefore, we coded overall evaluation> positively highlighted> breaks.<br>-Being able to control the degree of confrontation with the topic is important (see overall evaluation > negatively highlighted)	

Note. Coding tree is based on n=16 coding units. Example quotes are displayed in blue. All interviewees who reported suicidal ideation during an interview distanced themselves from actual, action-oriented suicidality. The quotes were translated from German into English.
