# Supplementary material for: Evaluation of an online suicide prevention program to improve suicide literacy and to reduce suicide stigma: A mixed methods study
Source: PLoS One. 2023 Apr 28;18(4):e0284944. doi: 10.1371/journal.pone.0284944 (PMC10146514; doi:10.1371/journal.pone.0284944)
Supplement: Attachment — Submitted filename: Evaluation of an online suicide prevention program to reduce suicide stigma A mixed methods study.pdf [file pone.0284944.s011.pdf]

Evaluation of an online suicide prevention program to reduce suicide stigma: A mixed methods study.

The introduction: The topic is directly addressed in the introduction. A clear and concise description of the research problem is provided. The main variables of the research are clearly outlined. A scientifically sound formulation of the research problem.

In regard to the goals and the importance of research, there are specific, clear, and straightforward objectives underlying the research that have been formulated in a straightforward manner**.** There is no doubt that research plays a significant role in the field of specialization, and it is advantageous to use it in that area.

The study was based on a number of relevant studies that were incorporated. There are a number of previous studies that have been used as the basis for this study in order to provide additional insight and information.

Research methodology considers the study's objectives. The application of systematic methods has been done in accordance with scientific standards and guidelines. In this study, the results of the research are presented in a creative, organized, and accurate way. The findings of the study are supported by sound scientific principles. It was found that the study's results are supported by those found in prior research or theories of science. The conclusion clearly reflects the research objectives and is it novel.

In the field of study, recommendations were useful. The recommendations contribute to scientific knowledge in the field of specialization. Furthermore, they were relevant to the topic and problem of the study. In the research, references were used throughout and at the end of the project to provide a sound scientific foundation for the work. Throughout the paper, references are limited to those that are cited in the text as cited in the reference list. Moreover, the writing style engages the reader and creates a sense of creativity in the reader. The research shows that the parts of the study are interconnected in a clear way.

Finally, in view of the fact that the research was conducted in accordance with the journal's standards, I recommend that this paper be accepted in its form.
